# Dental Poverty

**Published:** 1905-01-15

**Authors:** G. S. Junkerman

**Affiliations:** Cincinnati


### DENTAL POVERTY.

BY G. S. JUNKERMAN, D.D.S., CINCINNATI.

The statements and deductions received from this paper are not to be used as evidence of the writer's low estimation of the practice of dentistry *as it should be,* but *as it is.* We do not expect this paper to be an agreeable one. Poverty rarely, if ever, is so. Lessons may be taught from the dark, as well as the light side of life, and the general custom in dental societies has been somewhat deviated from in this document in that it refrains from public self-aggrandizement and the portrayal of professional class distinction, thoroughly convinced that the entire profession must live as an integer or be buried in an unrecognized mass in potter's field.

The dentist produces nothing and must therefore be classified as ''genus spongia,'' his pabulum being the public or laity. The laity no doubt cannot understand how poverty and dentistry can be mentioned in the same train of thought, except in a retrovertive sense upon the patient who has been unfortunate enough to require and receive services from one of the exponents of the calling. By chance there may be some few dentists also who think that they see an existing incongruity between the two subjects. For the existence of these conclusions in the mind of the laity, we will neither condemn *it,* nor try to correct it, being satisfied that no good will accrue, until such a time when the profession awakes to a full appreciation of the fact that poverty does stalk triumphant in its ranks. To those who cannot or will not see the existing condition, it is needless to offer consolation. They are like the turtle who, rapped with a stone, protrudes his head and then withdraws it again to its shell in fancied security, although the alarm may have been the forerunner of an avalanche or mountain slide that will crush out its life.

Not all the logic of Plato could persuade us to the conclusion that justice holds sway in the dental profession of today. Practitioners grow gray in the service, and those accredited by general consent with being at the head of their calling are dying from year to year. What has their harvest been? Their last moments will be free, at least, from worry over the late decree of the Supreme Court declaring constitutional the direct inheritance tax law. It is possible that the inventory will be taken on the other side of the great divide? If there be any reward for these shining lights it will have to be a heavenly one, as examination of their wills usually reveals a plentiful lack of stocks, bonds, cash and realty. The estate may consist of a large stock of human gratitude, but I fear this ceases to be a tangible asset after the spark of life has gone out.

In our rational and lucid intervals we are unable to comprehend how anybody can follow a calling, especially dentistry, without at least the anticipation of a commensurate reward. We may love or should at least be contented with our occupation in life, but it is only congenial and pleasant to us as the remuneration derived therefrom permits us to satisfy the tastes that a professional gentleman should possess. It has been openly asserted in dental meetings that no thought of fee should be commingled with the operation. This does not accord with my views and I will be honest enough to assert that no two thoughts can be more harmoniously blended than a beautiful operation and a beautiful fee. I love to think of them together, and the more munificent the fee appears the more beauty and inspiration I can throw into my operation.

Poverty exists in other forms besides in the estates of deceased dental lights. In their living estates we find continual manifestations of it. Good dentists are men of refined and cultivated tastes. They must have books, pictures, fine furniture and fine houses. The uncultivated laborer has no tastes or desires for such luxuries. This artistic taste and with it the desire must be co-existent with the artistic dentist. Have any of you lately purchased a Rembrandt or a Murillo? Do your incomes keep pace in a reasonable degree with your desires? There may be a few of the above-mentioned turtles who think they can answer these questions in the affirmative. Their restricted horizons, however, confined by their narrow shells, are not sufficient evidence except by way of exceptions preponderant enough to prove the rule.

Poverty exists also in the appreciation shown by public for dental services. There are only three things in life valued above all else. *Life*, itself, first, which is allegedly in the keeping of the physician; property conserved by the lawyer, and lastly the soul by the clergy. If such a condition existed that either of these three possessions would be lost coincidentally with the last tooth, there would not be enough dentists in the entire United States to supply the dental services required in the State of Ohio alone. Dental services may be of vital importance and they may have some relation to life, property and immortality, but you have not yet convinced the public of the existence of such a condition and so in their sight you are poverty stricken in public appreciation.

Another line of poverty which we have in stock is that which pertains to our methods. We concede the fact that there are a great many very poor dentists in practice, but there would be many more so declared, if they were weighed in the proper balance. The public is very indulgent even over those things which they do not appreciate; consequently many dentists pass as experts because they seem to have good general results from their operations. Patients may be vitally depleted, and have great pain inflicted that results may be attained. The successful operation and the dead patient, however, have never appealed to me as evidence of good professionalism. Much of the dread and lack of appreciation and hatred toward dentists to-day exists from the following of such methods. You are poverty stricken in this phase of dentistry unless you have a system of rapid and painless operations. Love and appreciation cannot be inspired by the protracted use or abuse of clamps, separators and rubber dam. The good dentist cannot be constituted of good results alone any more than the human body can be made of water entirely, although its chief constituent is that substance. Good results procured by speedy, painless and comfortable operations is what makes the true, scientific dentist.

Our literature is suffering from starvation. We are poor in system as portrayed by college text-books. It is almost impossible to find any two that agree on a certain method. System is the first principle of organization, and organization prohibits disintegration. Without systematized methods any professional calling sinks to one of charlatanism. If our literature does not permit us to systematize the instructions to our college recruits, how can we expect the veterans who constitute our societies to maintain their positions under rigid fire. Our literature is better than it was some years ago, but good text-books are quite as scarce now as then.

From the above deductions, which you may mentally verify or disallow, we at least have arrived at the conclusion that dentistry is suffering from a lack of red corpuscles or an impoverished condition of the blood. This condition may be the result of a taint in the blood which prevents organization of new or healing of old tissue. The question that interests us most is, has dentistry an incurable disease? Does it occupy the strata that its constituents justify? You cannot change the spots of the leopard nor can you cut out and restore the specks in the decayed dental fruit.

One microbe can do more harm than a barrel of antiseptic can do good. I do not assert, I ask, is dentistry at its proper level? If any of you have discovered the secret of reconstruction I should like to know it. There is very little incentive for high class men to enter the profession. High requirements and long curriculums will not entice high-grade men. High colors do not deceive the judge of good textures. Men who spend so much time, money and brain force in acquiring a calling, will at least exact the promise of an opportunity for compensation sufficient to house their cultivation and refinement from public contempt. One disgrace to the profession can do more harm than a hundred of you can do good, and the standing of the profession is not what it should be to-day, being neither attractive to the good men it has or the good ones that it should have. The cause of this condition does not lie alone in one quarter, but the general impoverished condition of public appreciation, improper and incomplete operative and fee system, impoverished dental literature making a highly unattractive mass for high-minded, cultivated humanity.

The fee system, which so nearly concerns our salvation, is a subject of a very delicate nature and like all other subjects of importance, has two sides. Professional men should be men of high ideals, and it requires money to maintain these ideals. If you can fill the profession with men whose requirements are great it might seem to open the gates to the securing of high fees by the profession. On the other hand, if men enter the profession whose ideals are low, and who require little to supply their wants, the fees will be correspondingly low. No better example of this exists than in our own country in regard to the Chinese exclusion act, which was thought of enough importance to be enacted by our national legislative bodies in order to protect the American labor. The Chinese coolies, who can live like rats, would otherwise have driven to starvation our laboring men who are the bone and sinew of this great country. Wages have remained good and our people have been able to raise and educate their families to good citizenship and good womanhood and manhood. The other side of the question is quite as important. All classes and conditions are unexempt from the requirement of dental services and the poorer classes find themselves unable to pay fees commensurate with the services rendered. These people are quite as important to a stable government and their wants must be supplied. In law and medicine the requirements of these respective professions are supplied to the poor by salaried exponents appointed by the city and municipal governments for their agents. The doctor for the poor, and the lawyer for the undefended, are matters of daily history in all cities of our country. Is this another proof of the lack of public appreciation for dental services? I presume that this crying need does not exist in dentistry, as no doubt you are well aware that even the poorest will find a dentist still poorer who is willing and forced to work for such a tithe as may be extracted from the purses of these indigents.

If I have stated any facts in this paper, please do not blame me for them. If my glasses are not properly focussed and I do not see right, I wish that you would all help me to adjust them. If you believe with me, work with me, and then perhaps some day we will all be

> Like him who climbs with sturdy stride,
> Far up the mountain's troubled side;
> And then turns round to look below,
> At hardships past, and conquered woe:
> So hearts by troubles past grow strong,
> And courage take in suffered wrong,
> To work, and hope, then attain that rest
> In nature's home on sunlit crest.

DISCUSSION.

Dr. W. A. Price, Cleveland: I would take exception to a few points in order that I may express my own views. He says we may live and should at least be content with our occupation in life, but it is only congenial and pleasant to us as the remuneration and profit permit us to satisfy the taste which professional gentlemen should possess. He also says poverty exists also in the appreciation shown by the public for dental services. First putting it on a financial basis in proportion to the fee and then poverty exists in the appreciation of the profession. "There is very little incentive for high-class men to enter the profession." And then he says that not all the logic of Plato could persuade us that justice holds sway in the dental profession. "Good results secured by speedy, painless and comfortable operations is what makes the scientific dentist." Now, what is an endowed dentist? What is a successful dental practice, a successful life as a dentist? We are probably going to divide right here where we start in. If we are to agree with our esteemed essayist, that the fee and appreciation of the profession make the success, then what of the men who have made the greatest contributions to science, literature and the arts, that never earned enough to pay for a week's board and never got an expression of appreciation during their life-time, and yet stand out as glorious characters? Gentlemen, we must take something else as a fundamental principle for successful life. Dr. Junkerman has said there are three things in life wanted above everthing else. Life itself, which is in the keeping of the physician; property, which is conserved by the lawyer; and lastly, the soul which is referred to the clergy. Lastly, No! First, the soul. What are we here for if not the good we can do for others. If life is going to be a success in dental practice it is only going to be a success so far as it loses sight of self for others. If we are going to be altogether selfish, we are going to be miserable. Why is it that we want the approval of the people for whom we work? It is simply a question of nature. A man naturally yearns for commendation. Now, then, do we get the commendation? We all, as dentists, have a personal experience, is it varied? Indeed it is varied. It is as varied as the men who go into the profession. He struck the key-note of the whole thing when he said, "good results secured by speedy, painless and comfortable operations is what makes the scientific dentist," and he should have added the appreciated dentist. If we are not going to be poverty-stricken, we must give high value. When a man tells me that the public does not appreciate dentistry, I simply say he does not know, for the public will express appreciation. A successful operation is only ideally successful when it is perfect from the standpoint of mechanical operation and free from pain to the patient.

Now, then, regarding the fee. Is the dental profession poverty-stricken for fees? I am afraid, as a profession, it is. He very aptly states when he starts it that he does not want this paper to be used as the writer's low esteem of dentistry as it should be, but as it is. But as he carries out the paper he does not give the remedy but simply leaves us at the close of the paper with the thought that good men will not enter the profession because there is no attraction for them. There is not a calling in the world that will give a man more pleasure in its practice, if he is full of the love of it, than the dental profession; but if he is in it for the dollars he can make out of it, he will always be poverty-stricken. The operator's heart has got to go out in sympathy in a way that it cannot do for dollars. I want to say again that there are men in this room whom I know get appreciation and expressions of appreciation both in words and fee that does repay them to their satisfaction for the operations they have performed. At the same time the great masses are not given that expression of appreciation, but they are architects of what they are building. I think the fee we get is about what we deserve. If we are not giving a very valuable service and appreciating it highly ourselves, we are not going to ask a high fee, and if we do not think it is worth a good deal, certainly we cannot expect our patients to. I do not wish to be unkind to a kind friend from another city, but I say the premises of the paper are wrong. There are four things to live for, not life, property and the salvation of the soul, but next to the salvation of the soul, the good we can do.—*Summary.*

